# Supplementary material for: ProteinShader: illustrative rendering of macromolecules
Source: BMC Struct Biol. 2009 Mar 30;9:19. doi: 10.1186/1472-6807-9-19 (PMC2672931; doi:10.1186/1472-6807-9-19)
Supplement: Additional file 1 — ProteinShader program without source code. This compressed file contains the complete ProteinShader program including associated libraries, but no source code. A README.txt file gives an overview of the ProteinShader distribution, and the index.html file in the help subdirectory has directions on getting started with the program as well as a set of tutorials. [file 1472-6807-9-19-S1.zip › ProteinShader-beta-0_9_4-binary/help/api/org/proteinshader/structure/factory/class-use/SegmentFactory.html]

Uses of Class org.proteinshader.structure.factory.SegmentFactory (ProteinShader API)


|  |  |  |
| --- | --- | --- |
| **Overview** \| **Package** \| **Class** \| **Use** \| **Tree** \| **Deprecated** \| **Index** \| **Help** | |  |
| PREV   NEXT | **FRAMES**    **NO FRAMES**     **All Classes** |  |


---


## **Uses of Class org.proteinshader.structure.factory.SegmentFactory**

| Packages that use SegmentFactory | |
| --- | --- |
| **org.proteinshader.structure** | Holds the classes that store information from a Protein Data Bank file: Structure, Model, Chain, AminoAcid, Heterogen, Water, Atom, Bond, Helix, BetaStrand, Loop, *etc*. |
| **org.proteinshader.structure.factory** | Holds the factory classes needed for generating the Segment objects that hold the information needed for rendering ribbon and tube segments. |

Uses of SegmentFactory in org.proteinshader.structure

| Methods in org.proteinshader.structure with parameters of type SegmentFactory | |
| --- | --- |
| `protected  void` | `Region.createSegments(SegmentFactory factory)`             This protected method should be called by the constructor of concrete subclasses of Region so that the Segment objects for the Region can be created with the appropriate type of SegmentFactory. |

Uses of SegmentFactory in org.proteinshader.structure.factory

| Subclasses of SegmentFactory in org.proteinshader.structure.factory | |
| --- | --- |
| `class` | `BetaStrandSegmentFactory`             Creates Segment objects for a BetaStrandSegmentFactory. |

---


|  |  |  |
| --- | --- | --- |
| **Overview** \| **Package** \| **Class** \| **Use** \| **Tree** \| **Deprecated** \| **Index** \| **Help** | |  |
| PREV   NEXT | **FRAMES**    **NO FRAMES**     **All Classes** |  |


---

# *Copyright © 2007-2008*
